# Deep learning models built from PSMA PET of the primary tumor can predict synchronous and metachronous prostate cancer metastases

**DOI:** 10.1371/journal.pone.0349825

**Published:** 2026-06-05

**Authors:** Jesus E. Juarez Casillas, Maryam Nezafat, Cecil M. Benitez, Kamil Rzechowski, Nathanael Kane, Karl Sjöstrand, Aseem Anand, Ida Sonni, Gholam R. Berenji, Sai Duriseti, Matthew B. Rettig, Nicholas G. Nickols

**Affiliations:** 1 Department of Radiation Oncology, David Geffen School of Medicine at UCLA, Los Angeles, California, United States of America; 2 Exini Diagnostics (subsidiary of Lantheus Holdings, Inc), Bedford, Massachusetts, United States of America; 3 Department of Radiation Oncology, VA Greater Los Angeles Healthcare System, Los Angeles, CA, United States of America; 4 Department of Radiology, David Geffen School of Medicine at UCLA, Los Angeles, California, United States of America; 5 Department of Medicine, VA Greater Los Angeles Healthcare System, Los Angeles, California, United States of America; 6 Department of Medicine, David Geffen School of Medicine at UCLA, Los Angeles, California, United States of America; 7 Department of Urology, David Geffen School of Medicine at UCLA, Los Angeles, California, United States of America; Universita Cattolica del Sacro Cuore, ITALY

## Abstract

**Objective:**

The objective was to develop prognostic models that included convolutional neural networks (CNN) derived from ^18^F-DCFPyL (PSMA) PET imaging of the primary tumor uptake patterns to prognose early metastatic progression after curative intent treatment for localized prostate cancer.

**Methods:**

Due to the lack of sufficient cases with adequate follow-up and metastatic events to derive this model directly, we derived models that predict the presence of synchronous metastases using only data obtained from the primary tumor. Because early metastatic progression events are consequent to occult metastases present at the time of initial therapy, we hypothesized that a model trained to predict synchronous metastases might also predict metachronous metastatic progression. A convolutional neural network (CNN) model was generated using whole-prostate PSMA PET images and auto-segmented intraprostatic lesions and combined with clinicopathologic data and imaging parameters to develop a multimodal model. Model performance was evaluated using the area under the receiver operating characteristic curve (AUC) for predicting synchronous metastases and metachronous metastatic progression.

**Results:**

The multimodal model and CNN model had AUCs of 0.82 (95% CI 0.69–0.92, p < 0.005) and 0.72 (95% CI 0.55–0.84, p 0.0059), respectively, for prediction of synchronous metastases. Shapley additive explanation analysis showed the CNN had the largest contribution to the combined model performance. For metastatic progression, the multimodal model had an AUC of 0.839 (95% CI: 0.6763–1.000, p = 0.0064).

**Conclusion:**

The multimodal model trained to predict synchronous metastases also predicted metachronous metastatic progression. This supports the potential of artificial intelligence applied to primary tumor PSMA PET images for enhanced prognostication. However, significant limitations include the modest sample size, single center source of data, and potential for overfitting, which may limit generalizability. Therefore, further validation in a larger cohort is indicated.

## Introduction

Prognostic assessments of localized prostate cancer can be derived from conventional clinicopathologic [1,2], transcriptomic [3], and digital histopathologic data [4]. In Prostate cancer, AI-based biomarkers and prognostic tools have been developed using data from digitized pathology slides and radiographic images.. Various AI models have been derived using slides from prostate needle biopsies to enhance diagnosis [5], prognostication [4,6,7], and therapeutic selection [8,9]. AI has similarly been applied to diagnostic prostate MRIs to predict recurrence after radical prostatectomy for localized prostate cancer [9–12]. PSMA PET/CT is now routinely used for staging patients with unfavorable intermediate- and high-risk prostate cancer [13]. AI has been applied to PSMA PET/CT for several purposes including estimation of ISUP grade group [14–16] and to identify [17], anatomically delineate, and quantify intraprostatic lesions [18,19]. AI tools have also been applied to PSMA PET/CT in the metastatic setting to prognosticate outcome after treatment for metastatic disease [20]. PSMA PET of the primary tumor may contain additional prognostic information amenable to machine learning to prognosticate outcomes after treatment for localized disease, which is the focus of the present study.

Our objective was to develop a model incorporating PSMA PET data from the primary tumor to prognose risk of metastatic progression after curative intent therapy for localized prostate cancer. However, we were limited in outcome data due to the relatively recent availability of PSMA PET imaging at our institution. We hypothesized that a model trained on PSMA PET imaging data within the volume of the prostate to predict synchronous metastatic disease could also identify patients with localized disease on PSMA PET at risk for early metastatic progression. This is hypothesized because most early metastatic progression events after curative intent therapy occur without evidence of local recurrence, suggesting growth of occult metastases present at the time of curative intent therapy are responsible for most of the early metastatic progressions [21]. We herein report development of a multimodal model derived from a convolutional neural network (CNN) model applied to whole-prostate ^18^F-DCFPyL (PSMA) PET images guided by auto-segmented lesions generated by the PSMA PET biomarker platform automated Prostate Cancer Molecular Imaging Standardized Evaluation (aPROMISE) [18,19], combined with clinicopathologic data and measurable imaging parameters that predicts synchronous metastases. We then sought to apply this model to predict metachronous metastases in a cohort of patients with localized prostate cancer after curative intent therapy.

## Materials and methods

**Fig 1:**
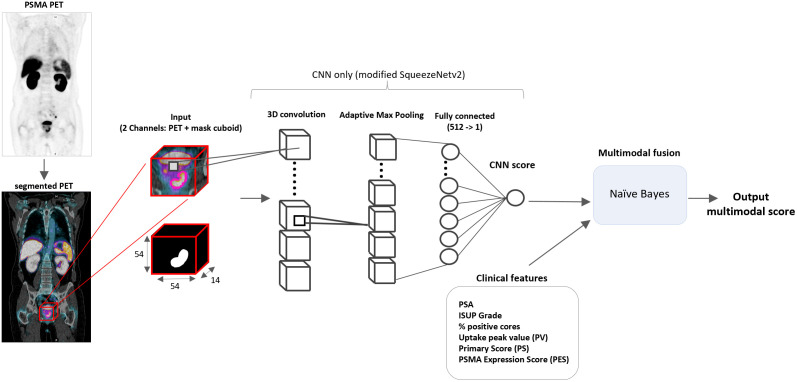
Model architecture. The DCFPyL PET images are first segmented using aPROMISE to delineate the prostate. A fixed-size 3D cuboid centered on the segmented prostate with the corresponding binary representation of the intraprostatic lesions is extracted and used as a two-channel input to a modified 3D SqueezeNetv2 mode (any metastases present were ignored). The output of the CNN -only model is obtained from the final fully connected layer as a single continuous score. For multimodal prediction, this CNN score is combined with clinical features using a Naïve Bayes classifier to generate the final multimodal output score. The multimodal score and CNN score had AUCs of 0.82 (95% CI 0.69-0.92, p < 0.005) and 0.72 (95% CI 0.55-0.84, p 0.0059), respectively, for prediction of synchronous metastases.

PSMA PET scans from 94 treatment naïve patients (S1 Table) obtained during initial staging at a single institution with standardized acquisition parameters were used for model development. This retrospective study was approved by the local institutional review board at Veterans Affairs Greater Los Angeles with a waiver of individual informed consent. The imaging showed either unequivocal evidence for metastatic disease (N = 45) or no metastatic disease (N = 49). The non-metastatic cohort subsequently completed curative intent therapy (radical prostatectomy or radiotherapy with androgen deprivation therapy) and had at least 33 months of follow-up without evidence of progression from the date of the scan. aPROMISE is an imaging biomarker platform developed specifically for PSMA PET/CT whole-body quantification and identifies and characterizes findings based on the PROMISE V2 framework [22]. A core feature of aPROMISE is the segmentation of the body into individual bone structures and other organs, including the prostate. aPROMISE was used to segment the entirety of the prostate and identify intraprostatic lesions while ignoring metastatic lesions. Prostate segmentation on the co-registered CT was used to map the PET image of the prostate. The PET image comprised a three-dimensional cuboid centered on the prostate. The PET image sub-portions containing the entire prostate and corresponding image volumes containing a binary representation of aPROMISE-defined intraprostatic lesions were used as inputs for the CNN (**Fig 1**). PET imaging artifacts or PET activity outside of the prostate were excluded by aPROMISE. This two 3D channel input (whole-prostate PET image and intraprostatic lesion map) was designed to guide the model’s attention toward areas of the prostate with the cancer while maintaining awareness of the entire prostate volume.

Several different CNN architectures were considered. Because of the limited number of training samples and the high risk of model overfitting, only those with a low number of parameters were considered, including ResNet18 [23], SqueezeNetv2, [24] MobileNet, [25] and ShuffleNet [26].

Squeezenetv2 was selected due to its compactness and associated low computational requirements. The architecture was adapted for volumetric image analysis by using 3D convolutions and pooling. All convolutional operations were implemented using 3D kernels of size 1x1x1 and 3x3x3 with padding applied to preserve spatial dimensions. For the CNN input, a cubic region of interest centered on the prostate area was extracted from the PET scan. Each input volume had a fixed size of 14x54x54 corresponding to the prostate area within the pelvic region. Images were normalized on a per-volume basis using min-max normalization to the range of [0,1]. Two input channels were used, the PET scan and corresponding hotspot representation. These channels were concatenated along the input channel dimension. PET and hotspots were concatenated along the channel dimension and fed to the CNN model. The base SqueezeNet architecture is described in [21].

The original SqueezeNet classifier (last Dropout, Convolution with kernel size 1, ReLU activation, and Average 3D Pooling layers) was replaced by Adaptive Max Pooling 3D (Output Size 1), Dropout (p = 0.8) and a Fully Connected layer mapping 512 features to a single output. The modified 3D SqueezeNet retained a lightweight design with approximately 1 million trainable parameters. Models were trained using the weighted Binary Cross Entropy loss function. The model was further optimized by performing grid-search hyperparameter-tuning. The optimal values for group convolution, learning rate, regularization strength, and set of augmentations were determined based on initial validation. We used a learning rate of 1e-4 with ReduceLROnPlateau scheduling. The neural network was trained for maximum of 300 epochs using early stopping with a patience of 140 epochs and the checkpoint for the best-performing epoch on the evaluation subset was kept for subsequent testing.

To avoid overfitting, a randomly selected set of augmentations was applied during training. At each epoch, a random subset of seven out of fifteen augmentations were selected and applied in random order exclusively to the training data. The augmentation pool included: random rotation, random flip in left-right axes, random Gaussian noise, random standard deviation intensity shift, random contrast adjustment, random Gaussian smooth, random Gaussian sharpen, random histogram shift, random Coarse shuffle, random 3D elastic distortion, random affine transformation, random Gibbs noise, random bias field, random K-space Spike Noise, and random Rician Noise. Random rotations were limited to +/-20◦ to preserve the prostate orientation as the model architecture consists of 3x3x3 convolutional kernels, which from design are not rotation invariant.

We also developed a combined (multimodal) model that added conventional clinicopathologic data (PSA, pathologic grade group, percent positive cores) and measurable imaging parameters (peak SUV prostate-located value, PRIMARY score, PSMA expression score per PROMISE-V2) [27,28] to the CNN via a Naïve Bayes classifier (**Fig 1**). A Naïve Bayes classifier was selected over other alternatives for its robustness in small, high-dimensional datasets, and compatibility with CNN-derived and clinical features. The dataset was split into training, validation, and test sets using stratified random sampling in ratios of 0.5, 0.2, and 0.3. In total, 94 patients (**S1 Table**) were included (Training: 47, validation:19 and test: 28). Model performance in predicting synchronous metastases was evaluated using the area under the receiver operating characteristic curve (AUC), with imaging-based ground truth as reference. Model training was repeated across 50 independent runs, and the best-performing model, as determined by AUC on the validation set was selected for final evaluation. The final CNN model and multimodal model were then tested to predict metachronous metastases in a cohort of patients with localized prostate cancer after curative intent therapy (**S2 Table**). Imaging and clinicopathologic data used in this project were retrospectively collected between May 4, 2023 and February 10, 2024. The authors that retrospectively accessed the data had access to information that could identify individual patients during data collection.

## Results and discussion

**Fig 2:**
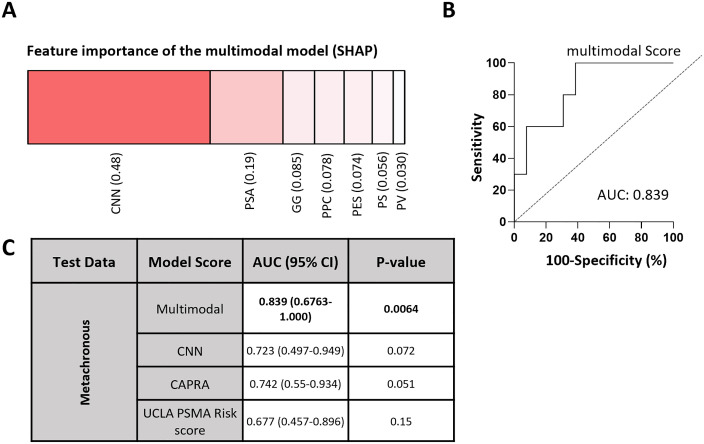
Performance of the multimodal model. **A.** Feature importance derived from Shapley additive explanation (SHAP) analysis showing the impact of contribution in descending order: CNN score, PSA value, pathologic grade group, percent positive cores, PRIMARY score, PSMA expression score, and peak PSMA uptake value. **B.** Receiver Operating Characteristic (ROC) curve of the multimodal model, with the area under the curve (AUC), **C.** Comparison of model performance, reported as AUC with 95% confidence intervals and corresponding p-values.

**Fig 3:**
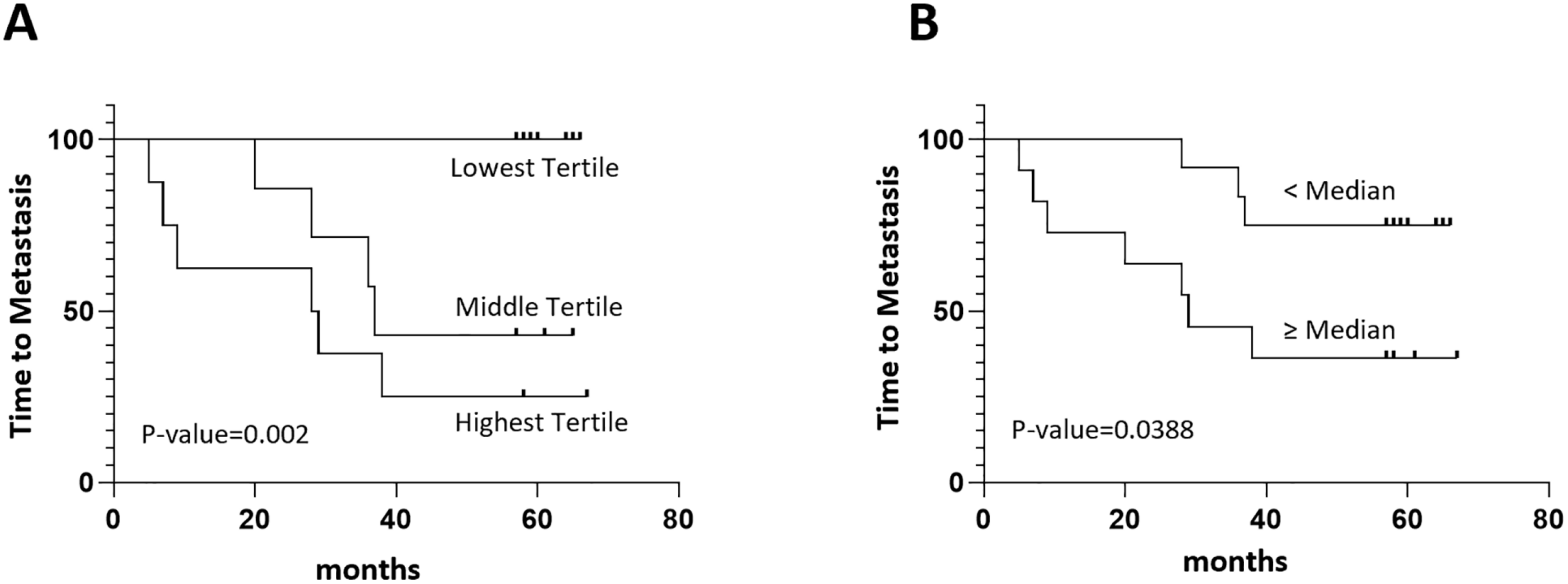
Kaplan-Meier survival analysis. Kaplan-Meier survival curves by multimodal score per tertile (**A**) and by median **(B)**.

The CNN and multimodal models achieved AUCs of 0.82 (95% CI 0.69–0.92, p < 0.005) and 0.72 (95% CI 0.55–0.84, p 0.0059), respectively, for synchronous metastases prediction (**Fig 1**). When considering each input independently, the CNN was the greatest contributing input based on Shapley additive explanation (SHAP) analysis (**Fig 2A**). Image based attention maps were generated using the FoxAI framework that demonstrates the model focuses attention on the regions of tumor within the prostate (**S1 Fig**). The models were applied to a cohort (n = 23) who had localized disease at initial PSMA PET, underwent curative intent therapy (radical prostatectomy or radiotherapy with androgen deprivation therapy), had at least four years follow-up, and had either no evidence of progression up to four years (n = 13), or metastatic progression by PSMA PET within four years (n = 10). Performance of metachronous metastases prediction was evaluated using receiver operating characteristics (ROC) analysis with discrimination quantified by the area under the ROC curve (AUC) and 95% confidence intervals calculated using DeLong’s method. The multimodal model achieved an AUC of 0.839 (95% CI: 0.6763–1.000, p = 0.0064), outperforming the CNN only model AUC of 0.723 (95% CI: 0.497–0.949, p = 0.072), the CAPRA score AUC of 0.742 (95% CI:0.55–0.934, p = 0.051) and UCLA PSMA Risk Score AUC of 0.677 (95% CI:0.457–0.896, p = 0.1538) (**Fig 2B-2C**). In summary, the CNN only model performed similarly to the CAPRA and UCLA PSMA Risk Score models that were comprised entirely of clinical features. The multimodal model, comprised of both the image-based CNN as well as clinical features, performed modestly better. Kaplan-Meier plots at tertiles and the median demonstrate differences in metastatic progression based on the multimodal score (**Fig 3**).

Our objective was to develop a prognostic model for early metastatic progression after curative intent therapy derived from PSMA PET data in the prostate. However, we lacked sufficient patients with adequate follow-up and metastatic progression events to train such a model directly. Instead, we developed a model that could predict for synchronous metastases by evaluating the PSMA PET data in the primary tumor. In training the model, non-metastatic cases were purposefully selected to minimize contamination from scans of patients who had early metastatic progression events and the metastatic cases were purposefully selected to minimize risk of false positives. Furthermore, a subset of patients with actual metachronous progression was withheld from model training and reserved for final model evaluation.

The multimodal model trained to predict synchronous metastases also predicted metachronous metastatic progression. This is consistent with the notion that occult metastases present at the time of initial curative intent therapy account for early metastatic progressions [21].

There are several limitations. One limitation to our study is that all scans used to develop the model were from a single center. This may limit generalizability. Moreover, the moderate size in the initial dataset resulted in a limited quantity of training samples, which limits model performance and narrows the options for model architecture. Given the case numbers used for model development, it is possible that reported AUCs are overestimates. Overfitting is another potential limitation. To address this, we employed models with a low number of parameters and implemented a random selection of augmentations for the CNN input. Another limitation is the small cohort used to test the model for metachronous progression which was severely limited by our access to images of patients who underwent PSMA PET with sufficient follow-up post treatment. These limitations render the findings as hypothesis generating and preliminary; the study is best interpreted as proof of concept. Accordingly, further validation in a larger multicenter cohort with additional performance metrics is necessary. A combined model of similar architecture but trained on a larger data set with actual cases of metastatic progression may have greater prognostic value.

## Supporting information

S1 Table Clinical pathologic features corresponding to the images that were used as inputs to train the model. Four scans did not have intraprostatic lesions identifiable by aPROMISE and were not used in the final CNN model. UIR = unfavorable intermediate risk, HR = high risk. *Diagnosed on metastatic biopsy and had no prostate biopsy.
(PDF)

S2 Table Clinical pathologic features of cases used to test the multimodal model prediction of metachronous recurrence. UIR = unfavorable intermediate risk, HR = high risk.
(PDF)

S1 Fig Attention maps. **A**. PET prostate-area cube. **B**. Intraprostatic lesions identified by aPROMISE. **C**. Attention maps using FoXAI.
(PDF)
