# Supplementary material for: Deep learning models built from PSMA PET of the primary tumor can predict synchronous and metachronous prostate cancer metastases
Source: PLoS One. 2026 Jun 5;21(6):e0349825. doi: 10.1371/journal.pone.0349825 (PMC13240907; doi:10.1371/journal.pone.0349825)
Supplement: S1 Table — Four scans did not have intraprostatic lesions identifiable by aPROMISE and were not used in the final CNN model. UIR = unfavorable intermediate risk, HR = high risk. *Diagnosed on metastatic biopsy and had no prostate biopsy. (PDF) [file pone.0349825.s001.pdf]

	Non-metastatic	Metastatic
NCCN Risk		
UIR	17 (35%)	6 (13%)
HR	32 (65%)	39 (87%)
cT Stage		
T1-2	31 (63%)	29 (64%)
T3-4	18 (37%)	16 (36%)
PSA		
Mean	14.78	79.13
Median	10.9	22.39
<10	21 (43%)	11 (24%)
10 to 20	19 (39%)	9 (20%)
>20	9 (18%)	25 (56%)
ISUP Grade Group		
1	3 (6%)	0 (0%)
2	14 (29%)	8 (18%)
3	14 (29%)	7 (16%)
4	12 (24%)	17 (38%)
5	6 (12%)	10 (22%)
n/a*	0 (0%)	3 (7%)
Percent Positive Cores		
≥50%	22 (45%)	30 (67%)
<50%	27 (55%)	12 (27%)
n/a*	0 (0%)	3 (7%)

Supporting Table S1. **Clinical pathologic features corresponding to the images that were used as inputs to train the model.** Four scans did not have intraprostatic lesions identifiable by aPROMISE and were not used in the final CNN model. UIR = unfavorable intermediate risk, HR = high risk. *Diagnosed on metastatic biopsy and had no prostate biopsy.
