# Supplementary material for: Deep learning models built from PSMA PET of the primary tumor can predict synchronous and metachronous prostate cancer metastases
Source: PLoS One. 2026 Jun 5;21(6):e0349825. doi: 10.1371/journal.pone.0349825 (PMC13240907; doi:10.1371/journal.pone.0349825)
Supplement: S2 Table — UIR = unfavorable intermediate risk, HR = high risk. (PDF) [file pone.0349825.s002.pdf]

	No Recurrence	Metachronous Metastatic Recurrence
NCCN Risk		
UIR	4 (31%)	2 (20%)
HR	9 (69%)	8 (80%)
cT Stage		
T1-2	6 (46%)	6 (60%)
T3-4	7 (54%)	4 (40%)
PSA		
Mean	10.95	23.25
Median	10.9	18.745
<10	6 (46%)	2 (20%)
10 to 20	5 (38%)	4 (40%)
>20	2 (15%)	4 (40%)
ISUP Grade Group		
1	1 (7.7%)	1 (10%)
2	4 (31%)	1 (10%)
3	6 (46%)	3 (30%)
4	1 (7.7%)	3 (30%)
5	1 (7.7%)	2 (20%)
Percent Positive Cores		
≥50%	8 (62%)	8 (80%)
<50%	5 (38%)	2 (20%)

Supporting Table S2. **Clinical pathologic features of cases used to test the multimodal model prediction of metachronous recurrence.** UIR = unfavorable intermediate risk, HR = high risk.
