# Supplementary material for: Deep learning models built from PSMA PET of the primary tumor can predict synchronous and metachronous prostate cancer metastases
Source: PLoS One. 2026 Jun 5;21(6):e0349825. doi: 10.1371/journal.pone.0349825 (PMC13240907; doi:10.1371/journal.pone.0349825)
Supplement: S1 Fig — A. PET prostate-area cube. B. Intraprostatic lesions identified by aPROMISE. C. Attention maps using FoXAI. (PDF) [file pone.0349825.s003.pdf]

A. PYL PET Image

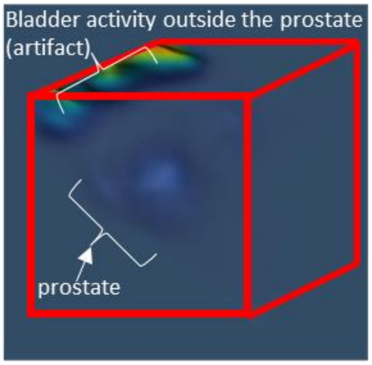

PET image within prostate centered cuboid

B. aPROMISE generated image

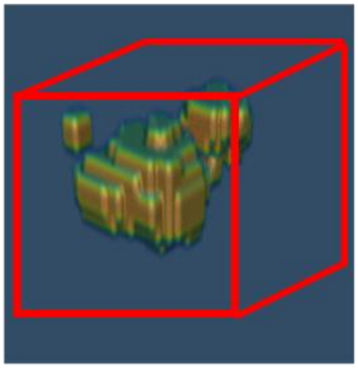

aPROMISE defined intraprostatic lesions

C. Attention map

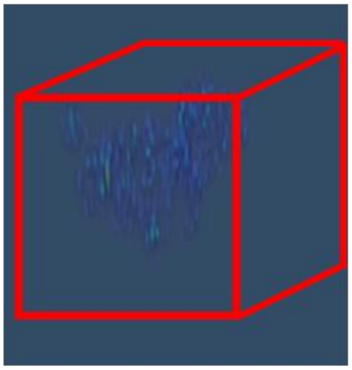

Image-based attention map using FoXAI

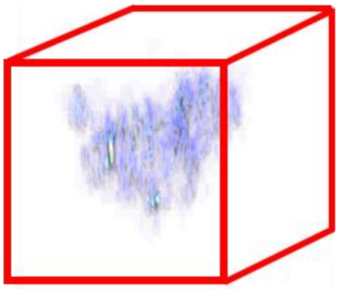

Image-based attention map using FoXAI (white background here for ease of visualization)

Supporting Fig S1. **Attention maps. A**. PET prostate-area cube. **B**. Intraprostatic lesions identified by aPROMISE. **C**. Attention maps using FoXAI.
